# Measuring the Protective Effect of Health Insurance Coverage on Out-of-Pocket Expenditures During the COVID-19 Pandemic in the Peruvian Population

**DOI:** 10.34172/ijhpm.2021.154

**Published:** 2021-11-07

**Authors:** Akram Hernández-Vásquez, Carlos Rojas-Roque, Antonio Barrenechea-Pulache, Guido Bendezu-Quispe

**Affiliations:** ^1^Centro de Excelencia en Investigaciones Económicas y Sociales en Salud, Vicerrectorado de Investigación, Universidad San Ignacio de Loyola, Lima, Peru.; ^2^Universidad de Buenos Aires, Buenos Aires, Argentina.; ^3^Universidad Científica del Sur, Lima, Peru.; ^4^Centro de Investigación Epidemiológica en Salud Global, Universidad Privada Norbert Wiener, Lima, Peru.

**Keywords:** Health Expenditures, COVID-19, SARS-CoV-2, Peru

## Abstract

**Background:** Health insurance coverage is expected to protect individuals from out-of-pocket (OOP) expenditures, potentially preventing them from falling into poverty. However, to date, the effect of health insurance on OOP spending during the coronavirus disease 2019 (COVID-19) pandemic has not been fully explored. This study aimed to estimate differences in the proportion and the amount of OOP expenditures among Peruvians during the pre- and post-mandatory lockdown response to COVID-19 in 2020 according to the health insurance coverage status.

**Methods:** This study utilized repeated cross-sectional data from the National Household Survey on Living and Poverty Conditions (ENAHO) from the first quarter of 2017 until the fourth quarter of 2020. The outcomes were (*i*) the proportion of individuals who incurred OOP expenditures and (*ii*) the monetary value of OOP expenditures. An interrupted time series analysis (ITS) and a quasi-experimental difference-in-difference (DID) analysis were performed to examine the outcomes among the control (individuals without health insurance) and treatment groups (individuals with health insurance) after the COVID-19 pandemic.

**Results:** ITS analysis showed that the proportion of individuals reporting OOP expenditures after implementation of mandatory lockdown due to COVID-19 in Peru decreased in both groups, but no difference in the slope trend was found (*P*=.916). The average quarterly amount of OOP spending increased in both groups, but no difference in the slope trend was found (*P*=.073). Lastly, the DID analysis showed that the mandatory lockdown was associated with a higher amount of OOP, but there was no evidence to indicate that the higher amount was different between the control and treatment groups.

**Conclusion:** The mandatory lockdown in response to the COVID-19 was associated with a higher amount of OOP expenditures and a lower likelihood of incurring OOP expenditures. However, our findings suggest that health insurance coverage does not lower OOP expenditures or reduce the likelihood of incurring OOP expenditures.

## Introduction

**Key Messages**

** Implications for policy makers**

- We found no difference in the mean amount of out-of-pocket (OOP) spending on healthcare between the population with and without insurance, before and after the mandatory lockdown due to the coronavirus disease 2019 (COVID-19) in Peru, suggesting that, on average, having health insurance in Peru does not significantly impact the amount of money spent on health as compared to not being insured.
- There was no difference in the proportion of people who incurred OOP spending both before and after the implementation of lockdown; thus, on average, individuals with insurance in Peru, still spend money on healthcare at a similar rate to those without insurance.
- After the implementation of lockdown, the mean amount of OOP spending increased compared to what was observed at baseline, suggesting that in Peru the implementation of this strict nationwide intervention left the population unattended and required them to spend additional resources to meet their healthcare needs.

** Implications for the public**

We found that after the implementation of a strict nation-wide lockdown the average amount of out-of-pocket (OOP) spending for healthcare increased by a similar amount among the general Peruvian population regardless of insurance status. This may have been predisposed by structural limitations in the healthcare system that are shared by many low- and middle-income countries and should be urgently addressed. These systems must be fortified and contingency plans for times of national health crises should ensure that the healthcare needs of the population are covered so that individuals do not have to resort to spending the limited resources that they have, placing them at risk of incurring catastrophic health expenditure.

The coronavirus disease 2019 (COVID-19) pandemic continues to produce a high burden of disease around the world. By June 21, 2021, a cumulative total of 179 743 520 severe acute respiratory syndrome coronavirus 2 (SARS-CoV-2) infections and 3 871 409 deaths attributable to COVID-19 had been reported worldwide.^1^ One of the measures proposed and implemented by many governments at the beginning of the pandemic to control SARS-CoV-2 transmission was mandatory quarantine. While this intervention, is useful for the control of the rapidly spreading viral pandemic caused by SARS-CoV-2, it implied a reduction or suspension of various economic activities. This scenario can lead to informal workers, whose financial status depends on their daily work income, being economically affected and leading many households to fall into poverty.

 Out-of-pocket (OOP) expenditures are direct payments made by individuals to healthcare providers for the use of medical services that are not reimbursed by any insurance, excluding any anticipated payment for the service.^2^ Having health insurance coverage is a measure that protects an individual economically when falling ill, as it reduces the upfront costs of healthcare and, in this way, reduces the probability of falling into poverty. However, the loss of job positions due to the recession caused by COVID-19 increased the number of people without healthcare coverage,^3,4^ raising the number of economically vulnerable people and resulting in catastrophic health expenditure in succumbing to COVID-19 or any other disease during the pandemic.

 Peru has been one of the countries most affected by the COVID-19 pandemic. By June 17, 2021, nearly a million COVID-19 positive cases had been detected with antibody tests (928 016), and 189 757 had died.^5^ As part of the disease control measures, in March 2020, a national state of emergency was established, which included a mandatory nationwide quarantine which caused a reduction in the mobility of people and the closing of non-essential productive activities.^6^ In Peru, most of the population (>70%) has an informal job. Between July 2019 and June 2020, informal employment increased from 72.6% to 74.3%.^7^ This scenario may indicate that an increasing number of people in this country faced disproportionate impacts on job and income losses due to the COVID-19 mandatory lockdown.

 Despite the growing economic and social crisis brought on by the COVID-19 pandemic, little has been studied regarding the effect it has had on OOP expenditures. Even though OOP expenditures constitute a part of the Peruvian health system financing, representing a significant percentage of the expenses made by Peruvian households,^8^ the impact of the pandemic on OOP expenditures has not been studied in this population. The economic impact caused by getting sick during the pandemic can affect the population as a whole and having health insurance coverage could help mitigate this impact. Thus, the objective of this study was to estimate differences in the proportion and the amount of OOP expenditures by Peruvians during the pre- and post-mandatory lockdown response to COVID-19 in 2020 according to health insurance coverage status.

## Methods

###  Study Population and Design 

 This study utilized repeated cross-sectional data from the National Household Survey on Living and Poverty Conditions (ENAHO, acronym in Spanish) from the first quarter of 2017 until the fourth quarter of 2020 (last available quarter) for a total of 16 quarters. ENAHO is a survey conducted by the National Institute of Statistics and Informatics of Peru (INEI, acronym in Spanish), which employs a stratified two-step independent probabilistic sample. Its objective is to collect information on the living conditions of the Peruvian population, and it is representative of the national, departmental, and natural region levels. ENAHO is conducted every quarter and allows obtaining estimations of the population applicable at national and urban/rural levels. This survey allows the aggregation of information to determine annual estimates.^9^

 ENAHO analyzes households and the habitual occupants in the rural and urban areas of the country. The unit of investigation is constituted by the members of the home, the workers of the household that sleep within the residence, whether they receive payment for their services or not, the members of a family pension that has a maximum of nine pensioners, and any individual that stayed in the household during the 30 days prior to the survey regardless of whether they are related to the other residents.^9^ On average, ENAHO surveyed, between 27 000 and 31 000 individuals per quarter.

 The ENAHO dataset is available in the quarterly and annual format. It can be freely obtained from the INEI website (https://www.inei.gob.pe/bases-de-datos/).

###  Measuring Out-of-Pocket Expenditures

 For this study, the main variables were the proportion of people who incurred OOP expenditures (yes/no) during the study period (first quarter of 2017 to the fourth quarter of 2020), and the amount of OOP expenditures measured in monetary values, disbursed by members of Peruvian households. A person who incurred OOP expenditures was defined as an individual who paid for healthcare that they or a member of the household received during the study period; this payment must not be covered by health insurance. Our definition included expenditures for over-the-counter drugs (drugs that do not require a prescription). People with OOP expenditures were assigned a value of 1 in the variable of interest (proportion of people making an OOP expenditure) and zero when this was not the case. It should be noted that up to 47% of Peruvians admit to purchasing at least one prescription medicine without a prescription, with this being especially common in areas with a lower supply of medical facilities.^10^

 The monetary value of OOP expenditures was constructed by summing the payments disbursed in nominal Peruvian soles for the care by or payment of any health service by the surveyed individual or a member of the household, including medical consultation, medication, laboratory or imaging exams, and other exams, dental health and other related services, ophthalmological services, paying for glasses, vaccines and health checkups, contraceptive medication, other expenses, hospitalization, surgical interventions and maternal health (control of pregnancy and delivery care). The reference period for disbursements (the recall period of the question regarding the OOP expenditures disbursed) was the last four weeks for medical consultation; the previous three months for dental and related services, ophthalmological services, child healthcare, and other health services; and the previous 12 months for hospitalization, surgical intervention, and maternal health. The monetary value of OOP expenditures was adjusted by inflation to the first quarter of 2021 using the consumer price index.^11^ OOP expenditures are reported in international dollars adjusted for purchasing power parity (PPP) in 2021^12^ to increase comparability with other countries.

###  Measuring Healthcare Insurance Status (Treatment Variable)

 Possessing healthcare insurance is the treatment variable in this study. The coverage by healthcare insurance was defined if the individual was affiliated with *Seguro Integral de Salud* (SIS; for the poor and extremely poor), or Social Health Insurance (EsSalud; for dependent workers and their legal beneficiaries), Police or Armed forces healthcare system, or being affiliated with a private health insurance provider. In Peru, of the total insured population, approximately 60% receive health services from the SIS; EsSalud treats 30% and the other healthcare providers, including the private sector, only provide services to 10% of the insured population^13^ Individuals not complying with this were defined as not having health insurance. For this study, people without health insurance coverage were considered the control group, while people with health insurance coverage were considered the treatment group.

 Regarding the overall insurance status of the Peruvian population, data from the 2017 national census indicates that approximately 24.5% of the total Peruvian population was still uninsured. According to age, 27.5% of the population aged 45-59 and 21.0% of those aged over 60 were not insured. Regarding area of residence 26, 5% of the population in an urban area were insured compared to 16.8% of those living in a rural area.^14^ As part of the Essential Health Insurance Plan implemented by the Peruvian government as of 2009, people who are insured with either SIS or EsSalud are not required to expend any additional money on medical consultation, laboratory, or imaging exams or medication for over 1400 diseases specified in the decree.^15^

###  Adjustment Covariables

 For the analysis, the following adjustment covariables were considered: gender (woman/man), age group (0-17 years/18-59 years/60 or more years), area of residence (urban/rural), educational level (none/primary/secondary/superior), and geographic domain (coast/highlands/jungle). The Coast region represents a narrow strip between the Western Cordillera and the Pacific Ocean, including Lima, the Peruvian capital. The Andean region is composed of a great variety of valleys, lakes, and pampas. The jungle region extends to both the Amazon plain and the eastern part of the Andes high forests.

###  Statistical Analysis 

 All analyses were conducted using the statistics software Stata v14.2 (Stata Corporation, College Station, Texas, USA). The complex characteristics of the sample design and weights of the survey were taken into account for all estimations using the command “*svy ”*. In all the analyses, statistical significance was evaluated with a *P* < .05, and the comparisons of the characteristics between the control and treatment group were performed using the *lincom* command in Stata. The command computes point estimates, standard errors, t or z statistics, *P* values, and confidence intervals (CIs) for linear combinations of coefficients after any estimation command, including survey estimation.

 Initially, an interrupted time series (ITS) analysis was conducted to evaluate the aggregate change in the variables of interest before and after implementing the mandatory lockdown in Peru (first quarter 2020). The ITS is a method used to evaluate changes in longitudinal series after a quasi-experimental intervention that occurs at a set point in time.^16,17^ The regression model of the ITS used in the analysis is based on the following equation:

*Y*_t_ *= β*_0_ *+ β*_1_ *T*_t_ *+ β*_2_ *X*_t_ *+ β*_3_ *X*_t_ *T*_t_ *+ β*_4_ *Z + β*_5_ *ZT*_t_ *+ β*_6_ *ZX*_t_ *+ β*_7_ *ZX*_t_ *T*_t_ *+ μ*_t_ (1)

 In equation 1, *Y*_t_ is the aggregate variable of interest measured every quarter *t*, *T*_t_ is the time, measured every three months that has passed since the start of the study (from quarter 1 to quarter 16), *X*_t_ is the intervention variable, which equals 1 in the quarters in which the mandatory lockdown was enforced and zero when not; *Z* is the treatment variable, *X*_t_*T*_t_, *ZT*_t_, Z*X*_t_, and Z*X*_t_*T*_t_ and are interaction terms. Thus, *β*_0_ is the average value of the variable of interest for the control group at the start of the study, while *β*_1_ is the value of the slope in the control group, *β*_2_ is the change in the slope of the variable of interest immediately after the intervention, *β*_3_ is the difference of the slope after and before the intervention, *β*_4_ the difference in the level between the treatment group and control group before the intervention, *β*_5_ is the difference in the slope between the treatment group and the control group before the intervention, *β*_6_ is the difference in the level between the treatment and control group in the period immediately after the start of the intervention and *β*_7_ is the difference in the slope (trend) between the treatment group and the control group after the beginning of the intervention compared with the pre-intervention. The coefficient *β*_7_ is akin to a difference-in-difference (DID) slopes, and hereafter this term will be referred to as “*difference in the slope trend*”. The term 𝜇_t_ represents the stochastic term of the regression.

 For both variables of interest, the stationarity of the time series were evaluated using the following unit root tests: the Levin-Lin-Chu test,^18^ the Harris-Tzavalis test,^19^ the Breitung-Das test,^20^ and the Im-Pesaran-Shin test.^21^ All tests have the null hypothesis that there is a single root in both groups (control group and treatment group). The 95% CIs of the parameters were estimated using Newey-West standard errors, which account for autocorrelation.^19^ The ITS analysis was performed using the Stata command “itsa,”^22^ and autocorrelation was evaluated using the Cumby-Huizinga test.^23^ Finally, the normal distribution of the residuals was assessed using the Shapiro-France test and the Shapiro-Wilk test.^24^

 Subsequently, changes in the amount of OOP expenditures before and after the first quarter of 2020 in individuals were further investigated using a quasi-experimental DID design. The DID methodology generally uses panel data to estimate the causal impact of policies or programs, although cross-sectional data are also used for the same purpose.^25-27^ For this analysis, the amount of OOP expense in the second, third, and fourth quarters of 2019 (time before mandatory lockdown) was compared to the second, third, and fourth quarters of 2020 (time after mandatory lockdown). The DID regression model is based on the following equation:

*Y*_t_ *= β*_0_ *+ β*_1_ *X*_t_ *+ β*_2_ *T*_t_ *+ β*_3_ *( X*_t_ * T_t_) *+ β*_4_*Z*_i_* + μ*_ti_ (2)

 In equation 2, *Y*_t_ represents the variable of interest, *X*_t_ is the treatment variable, the term* T*_t_ is the intervention variable that is zero in the second, third and fourth quarters of 2019 and is 1 in the second, third and, fourth quarters of 2020. The term (*X*_t_* T_t_)is an interaction between the exposure variable and the intervention, and* Z*_i_ is a vector of additional covariates of adjustment (adjustment covariables previously described) in the regression model. The term 𝜇_ti_ represents the stochastic term in the regression. Parameter *β*_0_ represents the initial level of the variable of interest during the study period, while parameter *β*_1_ represents the slope of the variable of interest up to the time of the intervention, parameter *β*_2 _represents the change in the level of the variable of interest that occurs after the intervention, parameter *β*_3_ represents the difference between the slopes of the result before and after the intervention, and parameter *β*_4_ represents the adjustment of the regression slope given the covariate included in the model.

 The DID was estimated with a generalized linear model with log link and gamma distribution to account for the skewed distribution of OOP amount values. With this model, estimates of DID in relative percentage changes and DID in absolute values estimated through the marginal effects of the predicted variable are reported, considering the interaction of the adjustment variables.

 Formally, the assumption of a parallel trend of the DID model was evaluated. The key assumption of the DID analysis is that the outcome of the treatment group (individuals with health insurance) would have had a similar trend to that of the control group (individuals without health insurance) in the absence of the intervention (mandatory social isolation). In other words, the control group serves as an appropriate counterfactual to the treatment group. To test this assumption, regression models were constructed with an interaction term between the treatment variable, a temporal measurement variable (continuous) before the start of the intervention, and the adjustment covariates if the coefficient of the interaction term was statistically significant (*P*< .05). This indicates that the linear trends between the treatment group and the control group differ during the reference period, suggesting a violation of the parallel trend assumption.^28^ Lastly, we formally assessed multicollinearity among the independent variables included in the model by performing the variance inflation factor (VIF). The highest VIF observed was <4.5. As there is no formal cutoff value to use with VIF for determining the presence of multicollinearity, the normal practice is to consider if VIF values exceed 10, it is often regarded as indicating multicollinearity problems.^29^ Therefore, our estimates indicate no problems of multicollinearity.

###  Sensitivity Analysis

 We conducted a sensitivity analysis of the DID regression model. First, the regression model was estimated using a different specification of the regression model (ordinary least squares specification). Second, adjusted regression models were estimated based on the hypothesis test through a thousand replications using the bootstrapping method.^30^ Third, we included data from a longer period to assess if a bigger sample size would affect our conclusion. Specifically, we included data corresponding to the second, third and fourth quarters of 2018 and 2019 and compared them to the second, third and fourth quarters of 2020.

## Results

###  Change in the Proportion of Out-of-Pocket Expenditures

**Figure 1:**
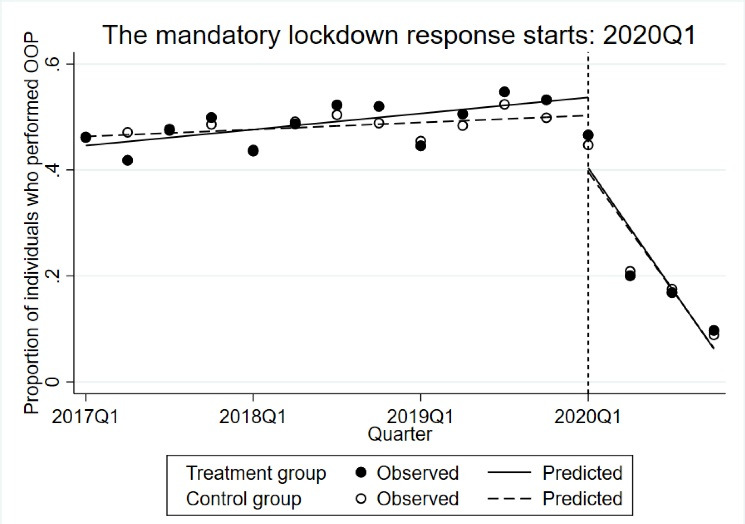
Quarterly Trend of the Proportion of Out-of-Pocket Expenditures Before and After the Mandatory Lockdown Response to Coronavirus Disease 2019 According to Health Insurance Coverage. Note. The treatment group corresponds to individuals with health insurance coverage whilst the control group corresponds to individuals without health insurance coverage. Abbreviation: OOP, out-of-pocket.

 Before the mandatory lockdown, a slight upward quarterly trend in the proportion of OOP was found in the control group (*P*= .001) but not in the treatment group (*P*= .084) (Figure 1). No differences were found in the proportion of OOP between the control group and the treatment group before lockdown (difference in proportion: 0.01, 95% CI: -0.01 to 0.04, *P*= .214). A significant reduction in the proportion of individuals with OOP expenses was found immediately after the mandatory lockdown and in the post-intervention quarterly trend. For the control group, the decline of the post-intervention trend was -0.11 (95% CI: -0.14 to -0.07, *P*< .001), and for the intervention group, the reduction of the post-intervention quarterly trend was -0.11 (95% CI: 0.15 to -0.06, *P*< .001). We found no difference in the slope trend of the proportion of OOP expenditures between the control and treatment group (-0.002, 95% CI: -0.05 to 0.06, *P*= .916).

###  Change in the Amount of Out-of-Pocket Expenditures

**Figure 2:**
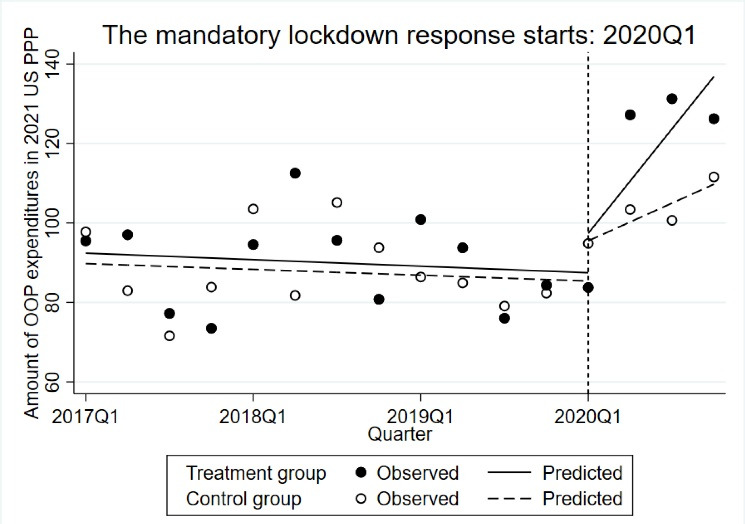
Quarterly Trend in the Amount of Out-of-Pocket Expenditures Before and After the Mandatory Lockdown Response to Coronavirus Disease 2019 Between Individuals With and Without Health Insurance Coverage in Peru. Note. The treatment group corresponds to individuals with health insurance coverage whilst the control group corresponds to individuals without health insurance coverage. Abbreviations: OOP, out-of-pocket; PPP, purchasing power parity.

 We did not find any quarterly trend in the amount of OOP expenditure for both the control and the intervention group (*P*= .925 and *P*= .658, respectively) (Figure 2). In addition, there was no difference in the amount of OOP expenses between the control and the intervention group during the pre-intervention period (*P*= .936). A significant increase in OOP expenditures was reported for both groups immediately after the mandatory lockdown and in the post-intervention quarterly trend. For the control group, the increase in OOP expenditures was US$ PPP 8.33 (95% CI: -0.65 to 17.32, *P*= .69), while in the intervention group, the rise in OOP expenditure was US$ PPP 19.98 (95% CI: 10.98 to 29.96, *P*< .001). We found no difference in the slope trend of OOP expenditures between the control and treatment group (US$ PPP 11.65, 95% CI: -1.06 to 24.35, *P*= .073).

 For the two main variables in the study, the formal tests performed to assess the unit root and the tests that evaluate the autocorrelation and normal distributions of the error terms showed no violation of the assumptions made (see Supplementary file 1).

###  Difference-in-Difference Estimates

 For the DID estimates before the mandatory lockdown (corresponding from the second quarter of 2019 to the fourth quarter of 2019), 69 878 individuals in the treatment group and 18 649 individuals in the control group were included. After the mandatory lockdown (from the second quarter of 2020 to the fourth quarter of 2020), 66 516 individuals were included in the treatment group and 12 184 individuals were included in the control group. The main characteristics of the population are described in Table 1. Most of the individuals in both groups were between 18-59 years old, lived in an urban area, and had secondary education. Most of the individuals were female except in the control group after the mandatory lockdown.

**Table 1:** Characteristics of Individuals Before and After Mandatory Lockdown Response to the COVID-19 Pandemic According to Health Insurance Coverage

**Characteristics**	**Before the Mandatory Lockdown Response to the COVID-19 Pandemic** ^a^			**After the Mandatory Lockdown Response to the COVID-19 Pandemic** ^b^		
	**Treatment Group (%) (n = 69 878)**	**Control Group (%) (n = 18 649)**	***P *** **Value** ^c^	**Treatment Group (%) ** **(n = 66 516)**	**Control Group (%) (n = 12 184)**	***P*** **Value** ^c^
Gender						
Female	48.45	44.37	<.001	48.56	54.83	<.001
Male	51.55	55.63	<.001	51.44	45.17	<.001
Age, in years						
0-17	35.96	20.89	<.001	35.77	21.78	<.001
18-59	51.84	70.75	<.001	51.45	70.77	<.001
60 or more	12.20	8.36	<.001	12.77	7.45	<.001
Place of residence						
Urban	75.78	88.14	<.001	76.2	88.49	<.001
Rural	24.22	11.86	<.001	23.8	11.51	<.001
Education						
None	11.93	6.61	<.001	11.55	5.39	<.001
Primary	28.87	19.98	<.001	28.77	19.67	<.001
Secondary	34.40	41.52	<.001	36.37	42.27	<.001
Higher	24.80	31.89	<.001	23.31	32.67	<.001
Geographic area						
Lima Metropolitan	31.02	36.34	<.001	31.01	37.33	<.001
Rest of the coast	22.84	25.68	<.001	23.17	24.88	.015
Highlands	32.09	27.91	<.001	31.91	28.20	<.001
Jungle	14.05	10.07	<.001	13.91	9.59	<.001

Abbreviation: COVID-19, coronavirus disease 2019. All the proportions are adjusted for the complex survey design of the ENAHO. The treatment group corresponds to individuals with health insurance coverage whilst the control group corresponds to individuals without health insurance coverage. ^a^ This period corresponds to the second quarter of 2019 to the fourth quarter of 2019. ^b^ This period corresponds to the second quarter of 2020 to the fourth quarter of 2020. ^c^ *P* values obtained with the *lincom* command in Stata. See the main text for further details.

 Although the treatment group showed a higher amount of OOP expenditure in comparison to the control group (a relative difference of 10%, equal to an absolute difference of US$ PPP 9.48), the DID estimation was not statistically significant (*P*> .05). These findings were qualitatively unaffected in the sensitivity analysis when a different model specification was performed and when the hypothesis was tested using the bootstrapping method or when we increased the sample size (Table 2). The formal test for the parallel trend showed evidence of parallel trends in both groups (Supplementary file 2).

**Table 2:** Change in the Amount of Out-of-Pocket Expenditures After Mandatory Lockdown Response to COVID-19 in Peru

**Amount of OOP Expenditures**	**DID Estimate (95% CI) **		***P *** **Value**	
	**Relative Percentage Change (%)** ^a^	**Absolute Change** ^a,b^	**Unadjusted**	**Adjusted** ^c^
2019 vs. 2020				
Generalized linear model, gamma family and log link	10.09 (-10.97 to 31.17)	$9.48 (-10.27 to 29.25)	0.347	0.349
Ordinary least square^d^	-	$17.32 (-6.00 to 40.64)	0.146	0.154
2018-2019 vs. 2020				
Generalized linear model, gamma family and log link	11.99 (-9.15 to 33.14)	$11.43 (-8.75 to 31.61)	0.266	0.264
Ordinary least square^e^	-	$15.60 (-4.00 to 35.19)	0.116	0.119

Abbreviations: OOP, out-of-pocket; COVID-19, coronavirus disease 2019; DID, difference-in-difference; CI, confidence interval. All the analyses are adjusted for the complex survey design of the ENAHO. ^a^ Adjusted for sex, age, place of residence, educational level and geographic area. ^b^ Estimated using marginal effects. See the main text for further details. US dollars adjusted for inflation to the first quarter of 2021 using the consumer price index and adjusted for PPP. See the main text for further details. ^c^ Hypothesis testing adjusted using the bootstrap method. ^d^ R-squared = 0.00132 and F-statistic = 35.44. ^e^ R-squared = 0.0100 and F-statistic = 54.59.

## Discussion

 Regarding the ITS analysis conducted in this study, we found that, the proportion of people that incurred OOP spending increased slightly throughout the 2017-2020 period, and decreased after the implementation of mandatory lockdown. Regarding the average quarterly amount of OOP spending, the relation was inverted; before the intervention, the average decreased every quarter, but after the intervention, the average amount of OOP spending increased in both those with and without health insurance coverage. Regarding the DID analysis, which compared the second, third, and fourth quarters of 2019 and 2020, there was no statistically significant DID in the mean amount of OOP expenditures between people with and without health insurance, suggesting that despite efforts to increase the coverage of healthcare insurance in recent years, health insurance coverage does not seem to protect against OOP expenditures.

 We observed a decline in the proportion of OOP spending in healthcare by the general population after the implementation of lockdown. The decrease in the utilization of primary healthcare and medical appointments has been documented around the world and has been related to the perception that the healthcare system was already overstretched, directives to self-isolate, and a general fear of the population contracting disease after visiting a healthcare center.^31,32^ Furthermore, a systematic review spanning 20 countries found that healthcare utilization decreased by approximately one third during the pandemic, primarily among individuals with less severe disease.^33^ In Peru, a military-enforced nationwide quarantine and interruption of all non-essential business practices were implemented in March 2020, aimed at halting the spread of COVID-19.^34^ Though it proved to initially reduce the reproductive number of the virus and enhance the resilience of the fragile health system, this drastic shift in the economy of millions of families, 70% of which depended on daily income from informal employment, likely led to the prioritization of all monetary resources towards ensuring the provision of food, housing, and essential goods.^35^ The extremity of the economic unrest led to the mass migration of thousands of families from the capital city to their hometowns on foot after losing their employment and closing their businesses.^36^

 After the onset of lockdown, the mean amount of OOP spending on healthcare increased among both those who were or were not insured. When SARS-CoV-2 reached South America, it quickly spread through the population and exceeded the capacity of many critical care health units that were not prepared for such a high demand. In counties such as Bolivia and Brazil the number of intensive care unit (ICU) would have to be multiplied by 60 and 12 fold to face the growing pandemic, respectively. While countries such as Guatemala and Haiti had little more than 100 mechanical ventilators between the two countries.^37,38^ The lack of resources also affected Peru, which likely incited OOP expenditures during the COVID-19 outbreak. During the COVID-19 pandemic, approximately 1600 ICU beds were available for the entire Peruvian population (33 million).^39^ The high demand and short supply of such a resource created an opportunity for criminal organizations within the public healthcare system to ask for approximately US$ 20 000 to ensure an ICU bed for a loved one in a reference hospital in Lima.^40^ Furthermore, in this country, about 85% of the oxygen supply was produced by a single company, leading to severe shortages and exponential increments in the cost of oxygen.^41^ This caused a rise in the price of a 10 m^3^ oxygen tank in the private market, from US$ 150 before the pandemic to upwards of US$ 1000. Likewise, a cubic meter of oxygen itself, which would typically last for only one day according to the severity of the disease, rose from around US$ 5 to about US$ 12.^42,43^ The expense derived from medical care and the cost of medicine and oxygen due to the saturation of the public health system, may have conditioned the OOP expenses of Peruvian households regardless of whether families had health insurance or not.

 The DID analysis did not find a statistically significant difference in OOP expenditures between those with and without healthcare insurance. This would suggest that being insured does not have an expected protective effect on OOP expenditures. A study conducted in 133 countries concluded that the proportion of the population covered by health insurance is a poor indicator of financial protection, and that countries should opt to increase the share of total health expenditure that is prepaid.^44^ Before the COVID-19 outbreak in Peru, up to 60% of people insured with SIS and over 90% of those insured with EsSalud reported OOP expenditures in the private health sector, suggesting that they could not find an adequate solution for their health problems within their health insurance system.^45^ In 2020 Peru rapidly opted to redirect all its limited resources towards the detection and treatment of the increasing number of COVID-19 cases leaving many chronically ill patients unattended.^34,46,47^ Like what was experienced in New Zealand, many non-urgent health needs, such as routine checkups and previously programmed surgical interventions were deferred or denied.^31^ In the capital of Peru, this gap led to an increase in the number of hospitalizations and deaths of previously well-managed patients with chronic illness and may have contributed to the rise in OOP expenses for habitual medication or additional spending to treat exacerbations of chronic disease regardless of the state of health insurance.^48^ Furthermore, many Latin American countries were subjected to misinformation spread through local news outlets and pseudoscientists on social media who claimed that diverse products cured or prevented COVID-19.^49,50^ Such events may have motivated many individuals to spend OOP money on various “preventive drugs” and may explain why up to 33% of patients hospitalized in a public Peruvian hospital had self-medicated prior to hospital admission.^51^ On the same note, it has been estimated that the price of essential drugs for the management of COVID-19 was 11 times higher in private compared to public pharmacies, with the purchase of therapy for severe cases (of COVID-19) in private pharmacies possibly requiring up to 64 days of the minimum daily wages.^52^ Hence, the COVID-19 pandemic may have led to a rise in OOP spending by individuals requiring care for other medical conditions regardless of their insurance status. Fake news and a general sense of powerlessness might have further contributed to investment of OOP money in miracle cures to protect family members.

 Among the limitations of this study, we should mention that it is not possible to establish causal relationships between study variables due to the observational nature of this study. Furthermore, since this survey relies on self-reported data by those surveyed, response and memory bias due to the sensitive nature of the questions being asked could be presented. Also, the survey questions related to healthcare-related expenditures may not fully represent real expenditure because of the different question periods. Additionally, it was not possible to evaluate the percent of income spent on OOP since the data source has no information about the income or total expenditure of a household. Likewise, because of the ecological approach to assessing the impact of health insurance on OOP expenditure on healthcare, we only considered the most broadly available forms of healthcare insurance which do not consider any additional benefits provided by a specific healthcare plan. Lastly, other variables that may affect the amount of OOP expenditures such as the health status of the individual, chronic diseases status and employment status were not included in the analysis. Despite these limitations, this study utilizes representative data collected to measure the socioeconomic conditions of the Peruvian population, which is widely used for decision-making in health and the analysis of socio-economic conditions and the population health status in this country. Therefore, the study of OOP expenditures in the Peruvian population using the data from ENAHO allows performing a good approximation of the study problem.

 In conclusion, after mandatory lockdown, there was a decrease in the proportion of Peruvians incurring OOP expenditures. However, the mean amount of OOP expenditures in health increased due to the COVID-19 outbreak. Despite the expected effect of health insurance, the proportion of people with OOP expenditures and the mean amount of OOP expenditures did not differ among those with and without health insurance coverage. Since having health insurance should protect the user against the expenses required for medical attention, the results of this study would suggest the absence of this protective effect. This lack of protection most likely responds to a combination of structural deficiencies in the health system and the interruption of non-urgent medical consultations as part of the containment measures to limit the spread of COVID-19. In the absence of a subsidized means of obtaining healthcare from saturated national health centers, individuals may have opted to spend OOP money to satisfy their healthcare needs. Thus, active steps toward integrating and fortifying the health system are needed to expand healthcare coverage and ensure that the needs of the population are covered to minimize the risk of OOP expenditure in the future. Future research may benefit from further analyzing if specific types of healthcare plans provide additional protection against OOP spending.

## Acknowledgments

 The authors are grateful to Donna Pringle for reviewing the language and style.

## Ethical issues

 Ethical approval was not required for this research due to the public and anonymous nature of the data we used.

## Competing interests

 Authors declare that they have no competing interests.

## Authors’ contributions

 AHV conceived the study. AHV and CRR collected the data and performed the statistical analyses. All authors interpreted the results, drafted the manuscript, and critically reviewed, and approved the final manuscript. All authors assume responsibility for the content of the manuscript.

## Funding

 Manuscript funding by Universidad Privada Norbert Wiener (obtained by GBQ) for management and analysis of data (CRR).

## Supplementary files

Supplementary file 1. Statistical Tests.

Supplementary file 2. Test for the Parallel Trend Assumption.
