# Supplementary material for: Measuring the Protective Effect of Health Insurance Coverage on Out-of-Pocket Expenditures During the COVID-19 Pandemic in the Peruvian Population
Source: Int J Health Policy Manag. 2021 Nov 7;11(10):2299–307. doi: 10.34172/ijhpm.2021.154 (PMC9808269; doi:10.34172/ijhpm.2021.154)
Supplement: Supplementary file 1 — Statistical Tests. [file ijhpm-11-2299-s001.pdf]

Article title: Measuring the Protective Effect of Health Insurance Coverage on Out-of-Pocket Expenditures During the COVID-19 Pandemic in the Peruvian Population

Journal name: International Journal of Health Policy and Management (IJHPM)

Authors' information: Akram Hernández-Vásquez[1]*, Carlos Rojas-Roque[2], Antonio Barrenechea-Pulache[3], Guido Bendezu-Quispe[4]

[1]Centro de Excelencia en Investigaciones Económicas y Sociales en Salud, Vicerrectorado de Investigación, Universidad San Ignacio de Loyola, Lima, Peru.

[2]Universidad de Buenos Aires, Buenos Aires, Argentina.

[3]Universidad Científica del Sur, Lima, Peru.

[4]Centro de Investigación Epidemiológica en Salud Global, Universidad Privada Norbert Wiener, Lima, Peru.

(*Corresponding author: ahernandez@usil.edu.pe)

Supplementary file 1. Statistical Tests

Unit-root tests for the proportion and amount of out-of-pocket expenditures series.

We performed a variety of tests to formally assess the stationarity (or unit-roots) for both the proportion and the amount of out-of-pocket expenditures. In each test performed, we included a linear trend in the model that describes the process by which the series is generated, and we removed the cross-sectional means. Each test performed has the null hypothesis that all groups (the control and the treatment group) contain a unit root.

Table S1. Unit-root tests for the proportion and amount of out-of-pocket expenditures series.

	**Proportion of out-of-pocket expenditures**	**Amount of out-of-pocket expenditures**
**Test**	***p*-value**	***p*-value**
Levin-Lin-Chu [1]	0.0016	<0.00001
Harris-Tzavalis [2]	0.0026	0.0001
Breitung-Das [3]	0.0015	0.0004
Im-Pesaran-Shin [4]	0.0145	0.0047

Cumby-Huizinga test for autocorrelation

We performed the test for autocorrelation in a time series proposed by Cumby and Huizinga [5]. The null hypothesis of the test is that the time series is a moving average of known order q, which could be 0 or a positive value. The alternative hypothesis states that serial correlation exists at specified lags >q.

Table S2. Cumby-Huizinga test for the proportion and amount of out-of-pocket expenditure series.

	**Proportion of out-of-pocket expenditures**	**Amount of out-of-pocket expenditures**
**Lags**	***p*-value**	***p*-value**
1	0.0544	0.8666
2	0.0652	0.0197*
3	0.9254	0.5019
4	0.7320	0.8319

*Since we found evidence of autocorrelation at lag 2, we corrected this by estimating a generalized linear square estimator with a population-averaged model specification, with link identity and Gaussian family and adjusting the correlation structure at lag 2.

Test to assess the normal distribution of the error terms

We performed the Shapiro-Wilk and Shapiro-Francia tests to assess the normality of the error terms [6]. The null hypothesis of both tests is that errors are normally distributed.

Table S3. Shapiro-Wilk and Shapiro-Francia test for the normality distribution of the error terms for the model using the proportion of out-of-pocket expenditure as a dependent variable.

	**Control group***	**Intervention group***
**Test**	***p*-value**	***p*-value**
Shapiro-Wilk	0.31592	0.12879
Shapiro-Francia	0.22967	0.06418

*The control group is represented by individuals without health insurance coverage while the intervention group is represented by individuals with health insurance coverage.

Table S4. Shapiro-Wilk and Shapiro-Francia test for the normality distribution of the error terms for the model using the amount of out-of-pocket expenditure as a dependent variable.

Test	Control group*	Intervention group*
	p-value	*p*-value
Shapiro-Wilk	0.34086	0.30078
Shapiro-Francia	0.27707	0.40631

*The control group is represented by individuals without health insurance coverage while the intervention group is represented by individuals with health insurance coverage.

Supplementary File 1 References

1. Levin A, Lin C-F, James Chu C-S. Unit root tests in panel data: asymptotic and finite-sample properties. J Econom [Internet]. 2002 May 1;108(1):1–24. Available from: https://www.sciencedirect.com/science/article/pii/S0304407601000987
2. Harris RDF, Tzavalis E. Inference for unit roots in dynamic panels where the time dimension is fixed. J Econom [Internet]. 1999 Aug 1;91(2):201–26. Available from: https://www.sciencedirect.com/science/article/pii/S0304407698000761
3. Breitung J, Das S. Panel unit root tests under cross-sectional dependence. Stat Neerl [Internet]. 2005 Nov;59(4):414–33. Available from: http://doi.wiley.com/10.1111/j.1467-9574.2005.00299.x
4. Im KS, Pesaran MH, Shin Y. Testing for unit roots in heterogeneous panels. J Econom [Internet]. 2003 Jul 1;115(1):53–74. Available from: https://www.sciencedirect.com/science/article/pii/S0304407603000927
5. Cumby RE, Huizinga J. Testing the Autocorrelation Structure of Disturbances in Ordinary Least Squares and Instrumental Variables Regressions. Econometrica [Internet]. 1992;60(1):185–95. Available from: http://www.jstor.org/stable/2951684
6. Royston JP. A Simple Method for Evaluating the Shapiro-Francia W’ Test of Non-Normality. Journal of the Royal Statistical Society Series D (The Statistician) [Internet]. 1983;32(3):297–300. Available from: http://www.jstor.org/stable/2987935
