# Supplementary material for: Measuring the Protective Effect of Health Insurance Coverage on Out-of-Pocket Expenditures During the COVID-19 Pandemic in the Peruvian Population
Source: Int J Health Policy Manag. 2021 Nov 7;11(10):2299–307. doi: 10.34172/ijhpm.2021.154 (PMC9808269; doi:10.34172/ijhpm.2021.154)
Supplement: Supplementary file 2 — Test for the Parallel Trend Assumption. [file ijhpm-11-2299-s002.pdf]

Article title: Measuring the Protective Effect of Health Insurance Coverage on Out-of-Pocket Expenditures During the COVID-19 Pandemic in the Peruvian Population

Journal name: International Journal of Health Policy and Management (IJHPM)

Authors' information: Akram Hernández-Vásquez[1]*, Carlos Rojas-Roque[2], Antonio Barrenechea-Pulache[3], Guido Bendezu-Quispe[4]

[1]Centro de Excelencia en Investigaciones Económicas y Sociales en Salud, Vicerrectorado de Investigación, Universidad San Ignacio de Loyola, Lima, Peru.

[2]Universidad de Buenos Aires, Buenos Aires, Argentina.

[3]Universidad Científica del Sur, Lima, Peru.

[4]Centro de Investigación Epidemiológica en Salud Global, Universidad Privada Norbert Wiener, Lima, Peru.

(*Corresponding author: ahernandez@usil.edu.pe)

Supplementary file 2. Test for the Parallel Trend Assumption

We formally tested the parallel trend assumption of the DID model. The key assumption of the DID analysis is that the outcome of the treatment group (individuals with health insurance coverage) would have had a similar trend to that of the control group (individuals without health insurance coverage) in the absence of the treatment (the mandatory lockdown in response to the COVID-19 pandemic)—i.e., the control group serves as an appropriate counterfactual of the treatment group were it not for the treatment. To test this assumption, we constructed the regression models with an interaction term between the treatment group indicator variable and year trend variable (as continuous) during the second and the fourth quarter (before the mandatory lockdown response). If the coefficient of the interaction term is statistically significant from zero, it indicates that the linear trends for expansion and non-expansion states differ during the baseline period, suggesting a violation of the parallel trend assumption.

Table S1. Test for parallel trend assumption

**Amount of out-of-pocket expenditures**	***p*-value**
Generalized linear model, gamma family and log link	0.324
Ordinary least square (OLS)	0.870
